# A novel dopamine receptor D2 antagonist (ONC206) potentiates the effects of olaparib in endometrial cancer

**DOI:** 10.1080/15384047.2023.2202104

**Published:** 2023-04-17

**Authors:** Sarah E. Paraghamian, Jianqing Qiu, Gabrielle M. Hawkins, Ziyi Zhao, Wenchuan Sun, Yali Fan, Xin Zhang, Hongyan Suo, Tianran Hao, Varun Vijay Prabhu, Joshua E. Allen, Chunxiao Zhou, Victoria Bae-Jump

**Affiliations:** a Division of Gynecologic Oncology, University of North Carolina at Chapel Hill, Chapel Hill, NC, USA; b Department of Obstetrics and Gynecology, the Second Hospital of Shandong University, Jinan, China; c Department of Gynecologic Oncology, Beijing Obstetrics and Gynecology Hospital, Capital Medical University, Beijing, China; d Chimerix, Durham, NC, USA; e Lineberger Comprehensive Cancer Center, University of North Carolina at Chapel Hill, Chapel Hill, NC, USA

**Keywords:** ONC206, olaparib, synergy, apoptosis, endometrial cancer

## Abstract

Poly ADP-ribose polymerase (PARP) inhibitors are effective therapies for cancer patients with homologous recombination (HR) deficient tumors. The imipridone ONC206 is an orally bioavailable dopamine receptor D2 antagonist and mitochondrial protease ClpP agonist that has anti-tumorigenic effects in endometrial cancer via induction of apoptosis, activation of the integrated stress response and modulation of PI3K/AKT signaling. Both PARP inhibitors and imipridones are being evaluated in endometrial cancer clinical trials but have yet to be explored in combination. In this manuscript, we evaluated the effects of the PARP inhibitor olaparib in combination with ONC206 in human endometrioid endometrial cancer cell lines and in a genetically engineered mouse model of endometrial cancer. Our results showed that simultaneous exposure of endometrial cancer cells to olaparib and ONC206 resulted in synergistic anti-proliferative effects and increased cellular stress and apoptosis in both cell lines, compared to either drug alone. The combination treatment also decreased expression of the anti-apoptotic protein Bcl-2 and reduced phosphorylation of AKT and S6, with greater effects compared to either drug alone. In the transgenic model of endometrial cancer, the combination of olaparib and ONC206 resulted in a more significant reduction in tumor weight in obese and lean mice compared to ONC206 alone or olaparib alone, together with a considerably decreased Ki-67 and enhanced H2AX expression in obese and lean mice. These results suggest that this novel dual therapy may be worthy of further exploration in clinical trials.

## Inroduction

Endometrial carcinoma (EC) originates from the epithelial cells of the endometrium and accounts for 90% of uterine cancers^1^. In 2022, there will be an estimated 65,950 new cases of endometrial cancer and 12,550 deaths in the US^2^. EC is one of the few cancers with both increasing incidence and mortality. This increase has been attributed to rising rates of obesity as obesity is a well-known driver of EC pathogenesis^3^. Obesity is associated with both higher risk of developing and dying from EC^3–6^. Based on the differences between molecular, clinical, and histopathological features, EC has been divided into two histopathological groups – type I and type II. Type I EC is estrogen-dependent, moderately differentiated to well-differentiated, surface-invasive endometrioid neoplasms that account for more than 70% of endometrial cancers. Type II EC is a non-estrogen-dependent, non-endometrioid, more aggressive growth with a high risk of recurrence and occurs in older women who are not obese^1,7^. While the prognosis is favorable for women who present with early-stage EC, survival rates for women with metastatic EC are less than 20%^8^. Thus, novel therapies are desperately needed for patients with advanced and recurrent cancer.

Poly(ADP-ribose) polymerase (PARP) has been implicated in DNA single-strand breaks, gene transcription and apoptosis^9,10^. PARP inhibitors are a class of drugs that inhibit PARP enzymes and, thus, prevent single-strand DNA break repair which leads to accumulation of double-stranded DNA breaks^10^. In tumor cells with BRCA mutations and/or deficient homologous recombination repair, PARP inhibition has greater impact on DNA repair, resulting in inhibition of tumor cell proliferation and induction of tumor regression^11,12^. While PARP inhibitors are FDA-approved as monotherapy in ovarian cancer, PARP monotherapy has not shown promising efficacy in clinical trials for EC^13^. Combining PARP inhibitors with agents that inhibit homologous recombination or with platinum-based agents as chemosensitizers may be effective strategies to increase the efficacy of PARP inhibitors in the treatment of EC^13,14^. This strategy has been successful in pre-clinical studies in multiple other tumor types^15–20^.

Imipridones are a novel class of cancer therapy that target dopamine receptor D2 signaling, induce TRAIL, and activate the integrated stress response via mitochondrial caseinolytic protease P (ClpP)^21–24^. ONC201, the first member of the imipridone class, is orally bioavailable and well tolerated^25^. ONC201 is currently being evaluated in numerous phase I and II clinical trials, including in EC. ONC206 is a derivative and chemical analogue of ONC201 that exhibits enhanced potency^26,27^ and is currently being evaluated in Phase I trials. Our recent study shows that ONC201 or ONC206 effectively inhibits tumor cell proliferation and tumor growth through inhibition of the Akt/mTOR pathway in EC cell lines and a transgenic mouse model of EC^28–31^.

PTEN and PIK3CA, which are frequently mutated in both type I (endometrioid) and type II (serous) ECs^32,33^, have been shown to play an indirect role in homologous recombination^15,16,34^. PI3K inhibition leads to downregulation of BRCA, and loss of homologous recombination results in increased DNA damage and sensitivity to PARP inhibitors^15,16^. The inhibitory effects of ONC201 and ONC206 on tumor growth are thought to be via their effects on the PI3K/AKT/mTOR pathway as well as DNA repair^21,29,35,36^, suggesting that ONC201 and ONC206 may be logical therapeutic partners for PARP inhibition in EC. Since PARP inhibitors and imipridones are both being evaluated in EC clinical trials but have yet to be explored in combination in pre-clinical models, the objectives of the present study were to determine the anti-proliferative effects of olaparib in combination with ONC206 in EC cell lines and a transgenic mouse model of endometrioid EC.

## Results

### Olaparib and ONC206 effectively inhibited cell proliferation and had synergistic anti-proliferative effects in EC cells

**Figure 1.**
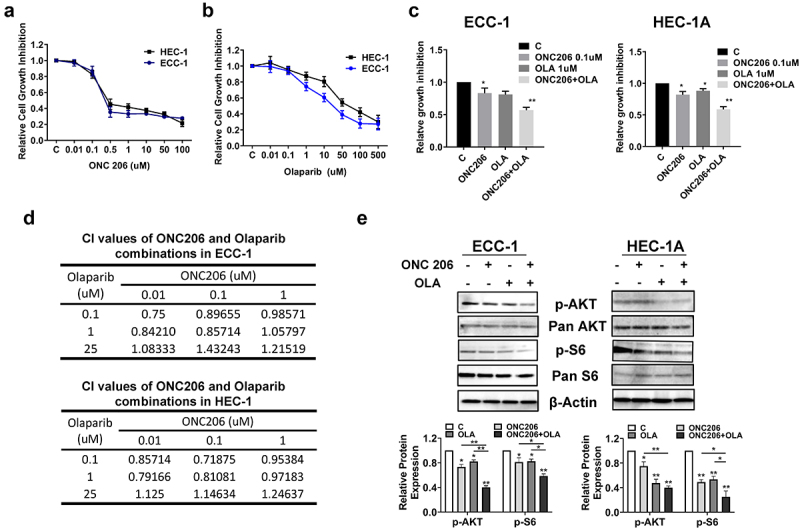
Synergistic effects of olaparib and ONC206 in EC cells. ECC-1 and HEC-1A cell lines were treated with varying doses of olaparib and ONC206 as single agents for 72 h. Cell proliferation was assessed by MTT assay. Olaparib and ONC206 significantly inhibited cell proliferation in a dose dependent manner (a and b). Simultaneous exposure to both drugs at low doses resulted in synergistic inhibition of cell proliferation in both cell lines (c). The combination index (CI) was calculated by the Bliss Independence model (d). Western blotting results indicated that ONC206, olaparib and the combination treatment reduced phosphorylation of AKT and S6 in both cell lines after 24 h of treatment (e). OLA is an abbreviation for Olaparib. (* = p < .05, ** = p < .01).

To investigate whether olaparib and ONC206 exhibits anti-proliferative activity against EC cells, we treated two EC cell lines, ECC-1 and HEC-1A, with different concentrations of olaparib and ONC206 for 72 h. Confirming our prior work, the MTT assay showed ONC206 potently inhibited cell proliferation in a dose-dependent manner in both cell lines with an IC50 of 0.19 µM for ECC-1 and 0.31 µM for HEC-1A (Figure 1A). A similar inhibitory effect was observed in olaparib treated ECC-1 and HEC-1A cells. Olaparib significantly suppressed growth of both cell lines in a dose-dependent manner with an IC50 of 8.64 µM for ECC-1 and 42.06 µM for HEC-1A (Figure 1B).

We next explored the efficacy of the combination of olaparib and ONC206 in EC cells. Co-treatment with different concentrations of olaparib and ONC206 resulted in greater inhibition of cell proliferation than with either drug alone after 72 h of treatment (Figure 1C). To determine whether the effects of simultaneous exposure were synergistic, the combination index (CI) was calculated using the Bliss Independence model. Synergistic anti-proliferative effects (CI < 1) were found with olaparib at doses of 0.1–1 µM and ONC206 at doses of 0.01–0.1 µM. In contrast, the combination of high doses (25 µM) of olaparib and varying doses of ONC206 did not induce synergistic reduction in cell viability (Figure 1D).

Given that imipridones inhibit cell proliferation and tumor growth through effects on the AKT/mTOR pathway in EC^36^, we next investigated whether the AKT/mTOR pathway was involved in the synergistic anti-proliferative effects of olaparib and ONC206 in the EC cell lines. Both cell lines were treated with ONC206, olaparib, and the combination for 24 h. Western immunoblotting results show that olaparib and ONC206 significantly inhibited phosphorylation of AKT and S6. Dual therapy resulted in even greater effects on reducing phosphorylation of AKT and S6 in both cell lines (Figure 1E). Together, these results suggest that the combination of olaparib and ONC206 synergistically induced growth inhibition through modulation of the AKT/mTOR pathway in EC cell lines.

### The combination of olaparib and ONC206 effectively induced apoptosis in EC cells

**Figure 2.**
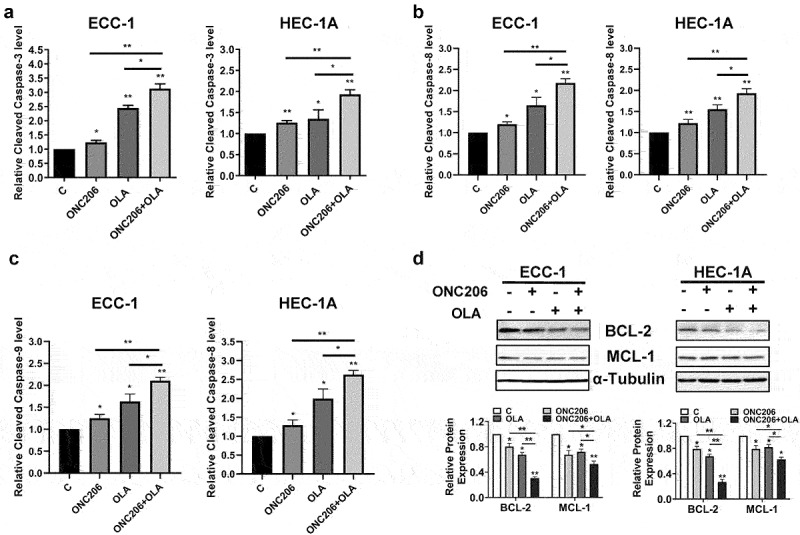
Olaparib and ONC206 induced apoptosis in EC cells. ECC-1 and HEC-1A cells were treated with 0.1 µM ONC206, 1 µM olaparib and the combination of olaparib and ONC206 for 12 h. Olaparib and ONC206 increased cleaved caspase-3 activity in both cell lines with greater effects for the combination compared to either drug alone (a). Western immunoblotting demonstrated decreased expression of the anti-apoptotic proteins BCL-2 and MCL-1, with olaparib and ONC206 treatment (b). OLA is an abbreviation for Olaparib. (* = p < .05, ** = p < .01).

Olaparib and ONC206 have been known to both induce apoptosis in EC cells^27,37^. To determine the effect of co-treatment with olaparib and ONC206 on apoptosis in EC cells, an ELISA cleaved caspase-3 assay was performed after treatment with ONC206, olaparib, and the combination to evaluate the effect of treatment on cleaved caspase-3 activity. Treatment with ONC206 (0.1 µM) effectively increased cleaved caspase-3 activity by 1.23 times in the ECC-1 cells and 1.26 times in the HEC-1A cells compared to controls, respectively. Olaparib at 1 µM increased cleaved caspase-3 activity by 2.45 times in the ECC-1 cells and by 1.35 times in the HEC-1A cells. The combination of both drugs led to a greater effect compared to either drug alone in both cell lines (Figure 2A). Consistent with the results of cleaved caspase-3, ONC206, olaparib, and the combination exhibited similar effects on cleaved caspase-8 and −9 activities in both cell lines, with the combination treatment producing the greatest effects (Figure 2B-C). Western immunoblotting demonstrated decreased expression of the anti-apoptotic proteins BCL-2 and MCL-1 in both cell lines that was further enhanced by combination treatment, mirroring the caspase-3 assay in the ECC-1 and HEC-1A cells (Figure 2D). These results suggest that induction of apoptosis by olaparib, ONC206, or the combination may be dependent on caspase pathways in EC cells.

### The combination of olaparib and ONC206 effectively increased cellular stress in EC cells

**Figure 3.**
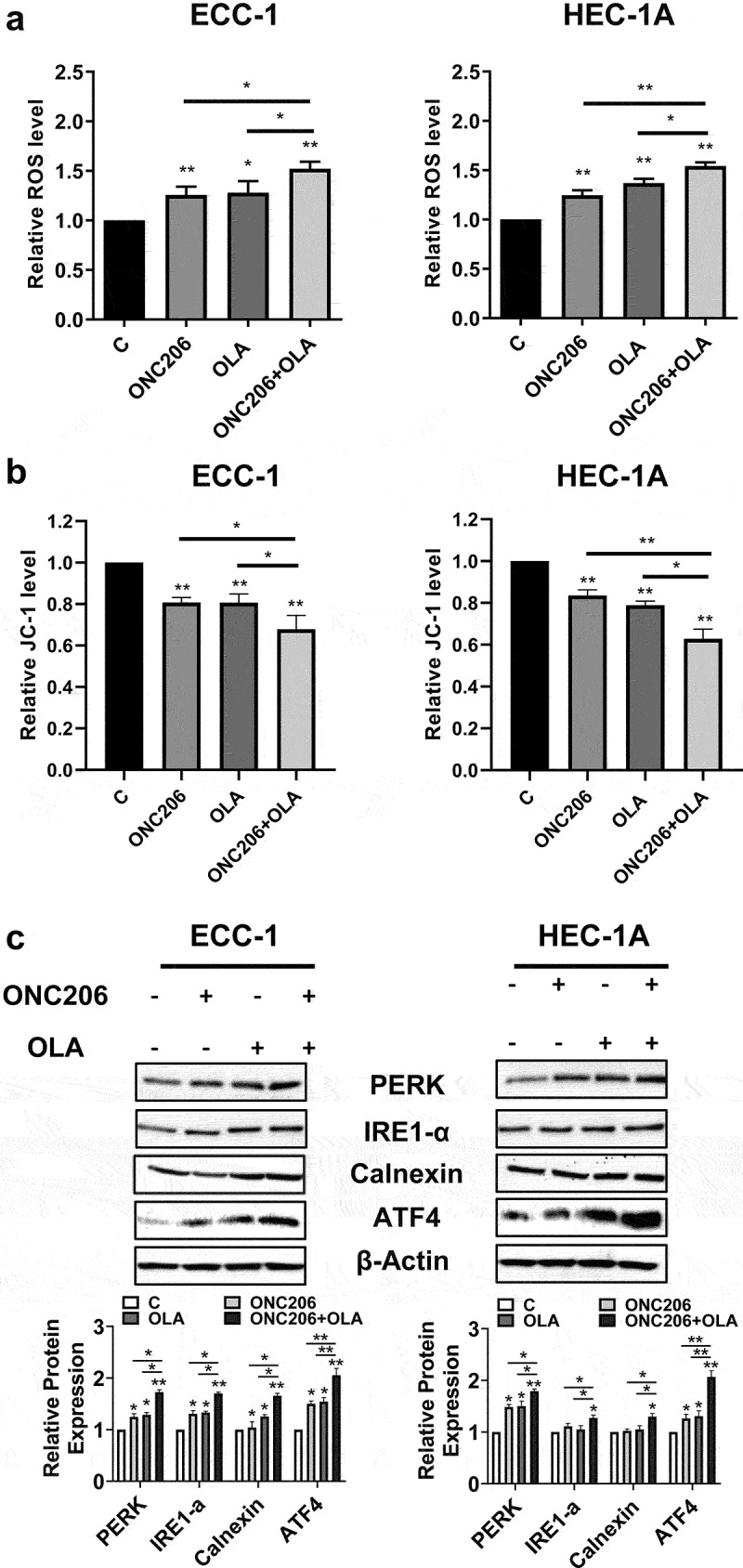
Olaparib and ONC206 induced cellular stress in EC cells. ECC-1 and HEC-1A cells were treated with 0.1 µM ONC206, 1 µM olaparib and the combination. ROS was assessed by DCFDA assay. Combination therapy with olaparib and ONC206 resulted in increased reactive oxygen species and mitochondrial membrane potential as compared to either drug alone (a and b). Confirmatory western blot assays demonstrated increased expression of the cellular stress proteins PERK, IRE, ATF4 and calnexin with combination treatment (c). OLA is an abbreviation for Olaparib. (* = p < .05, ** = p < .01).

Given evidence that imipridones activate the integrated stress response^25,27^, we examined the effects of olaparib and ONC206 on production of reactive oxygen species (ROS) using the DCFH-DA assay. ONC206 (0.1 µM) treatment resulted in a 1.25-fold increase in ROS in the ECC-1 cells and a 1.31-fold increase in the HEC-1A cells. Olaparib (1 µM) treatment resulted in a 1.28-fold increase in the ECC-1 cells and a 1.37-fold increase in the HEC-1A cells. Combination olaparib and ONC206 therapy led to a 1.5-fold increase in ROS over controls in both cell lines, which was also significantly increased compared to either drug alone (Figure 3A). Similar results were observed via the JC-1 assay. ONC206 or olaparib reduced mitochondrial membrane potential with the greatest effects seen after combination treatment in both cell lines (Figure 3B). Confirmatory western blots demonstrated increased expression of the cellular stress proteins PERK, ATF4, IRE, and calnexin with ONC206, olaparib, or combination treatment in both cell lines after 16 h of treatment (Figure 3C).

### *The combination of olaparib and ONC206 reduced tumor weight* in vivo

**Figure 4.**
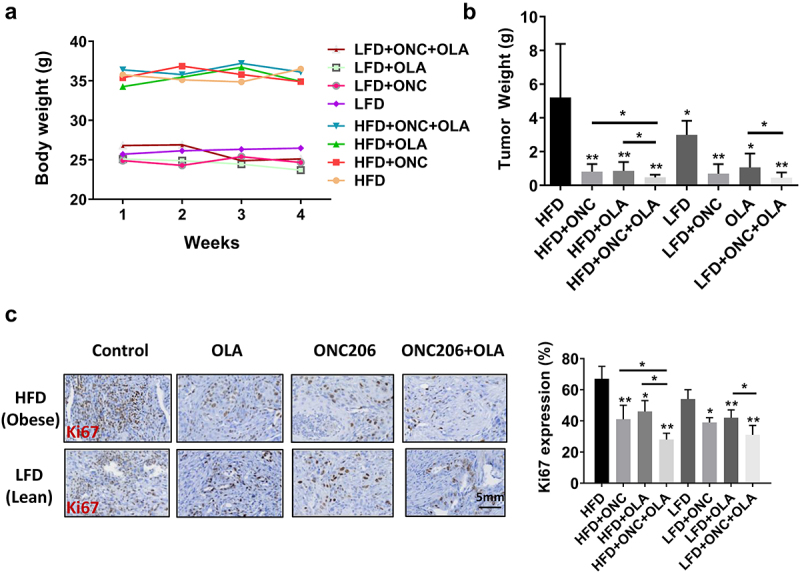
Synergistic effect of treatment with olaparib and ONC206 in vivo. Lkb1^fl/fl^p53 ^fl/fl^ mice were fed HFD (obese) or LFD (lean) starting at 3 weeks of age. After injection of AdCre into one uterine horn at 8 weeks, the mice were treated with ONC206 (100 mg/kg weekly), olaparib (25 mg/kg daily) and the combination for 4 weeks. Diet-induced obesity significantly increased the body weights of Lkb1^fl/fl^p53 ^fl/fl^ mice (a). Olaparib and ONC206 effectively reduced tumor weights, and combination treatment led to a significantly greater reduction in tumor weight compared to either drug alone under both obese and lean conditions (b). Immunohistochemistry showed that olaparib and ONC206 significantly reduced expression of Ki-67, a marker of tumor cell proliferation, in both obese and lean mice, with more potent effects seen with combination treatment (c). OLA is an abbreviation for Olaparib. (* = p < .05, ** = p < .01).

In order to further verify the inhibitory effect of ONC206, olaparib, and the combination *in vitro*, we treated the *Lkb1*^*fl/fl*^*p53*^*fl/fl*^ genetically engineered mouse model of EC with ONC206, olaparib, and the combination of both drugs for 4 weeks under obese and lean conditions (15 mice/group). Obese mice weighed significantly more at the start of treatment compared to lean mice (35.7 g in obese vs 25.6 g in lean, *p* < .01). During the varying treatments, all mice exhibited normal activities with no changes in body weight, and both drugs were well tolerated individually and in combination in the mice (Figure 4A). As seen in our prior studies using the same mouse model, obese mice had significantly greater tumor weights compared to lean mice across all treatment groups (5.29 g vs 2.98 g, *p* < .04) Treatment with ONC206 decreased tumor weight by 84% in obese vs 77% in lean mice. Olaparib decreased tumor weight by 83% in obese vs 65% in lean mice. The combination decreased tumor weight by 91% in obese vs 85% in lean mice. The reduction in combination treatment compared to olaparib monotherapy was significant for both obese and lean mice (*p* < .05). The reduction in combination treatment compared to ONC206 monotherapy was significant for obese mice only (*p* < .05, Figure 4B). Immunohistochemistry showed that olaparib and ONC206 significantly reduced expression of Ki-67, a marker of tumor cell proliferation, in both obese and lean mice, with more potent effects with combination treatment (Figure 4C).

### The combination of olaparib and ONC206 resulted in a greater inhibitory effect on DNA repair

**Figure 5.**
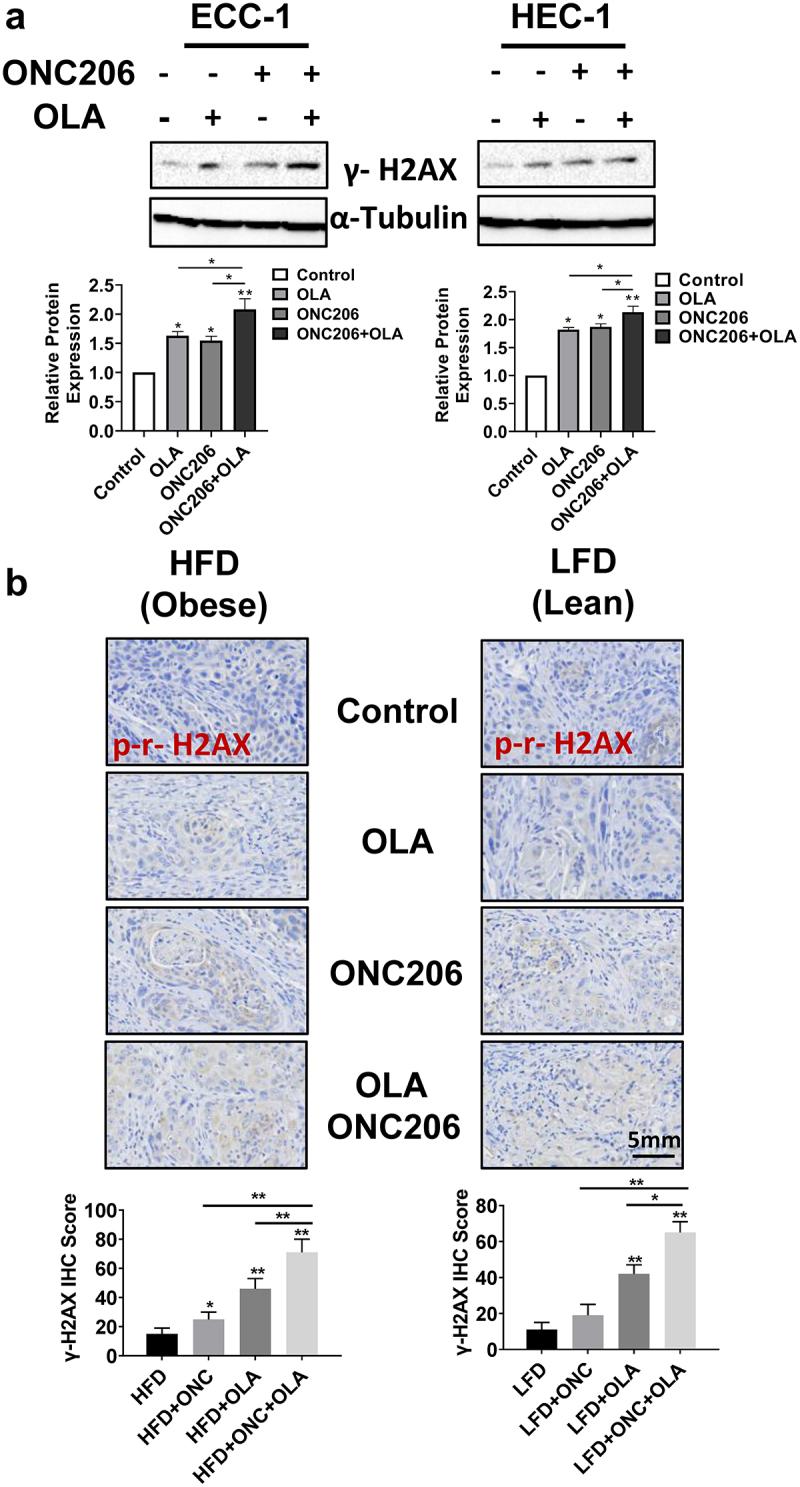
Olaparib and ONC206 induced the expression of phosphorylated γ-H2A× in vitro and in vivo. ECC-1 and HEC-1A cells were treated with 0.1 µM ONC206, 1 µM olaparib and the combination for 24 h. Western blotting showed that olaparib and ONC206 increased the expression of phosphorylated γ-H2A× in both cell lines with greater expression in the combination treatment (a). The expression of phosphorylated γ-H2A× in tumor tissues was detected by immunohistochemical staining after Lkb1fl/flp53 fl/fl mice were treated with ONC206, olaparib and the combination for 4 weeks. Dual therapy with olaparib and ONC206 increased gamma-H2A× as compared to treatment with either drug alone in obese and lean mice (b). OLA is an abbreviation for Olaparib. (* = p < .05, ** = p < .01).

In order to better understand how ONC206, olaparib and the combination treatment affect DNA repair, we determined the protein expression of phosphorylated γ-H2A× following treatment with ONC206, olaparib, and the combination *in vitro* and *in vivo*. Western blotting results showed that after treatment with olaparib and ONC206 for 24 h, levels of phosphorylated γ-H2A× (a marker of DNA damage) were increased in both the ECC-1 and HEC-1A cells compared with vehicles. Furthermore, phosphorylated γ-H2A× levels were most pronounced with the combination of olaparib and ONC206 treatment in both cell lines (Figure 5A). To confirm the immunoblotting results in EC cells, the expression of phosphorylated γ-H2A× was examined with immunohistochemistry in endometrial tumors under obese and lean conditions. Single agent olaparib and ONC206 treatments increased phosphorylated γ-H2A× in the tumors of obese and lean mice. Combined olaparib and ONC206 treatment significantly enhanced phosphorylated γ-H2A× expression over that of either drug alone (Figure 5B).

## Discussion

Given the poor prognosis associated with advanced and recurrent EC and the steady rise in mortality from EC, there is a critical need to develop novel therapies for patients with EC. Olaparib as a single agent or as a chemosensitizer has shown promising clinical efficacy in preclinical models and clinical trials in multiple types of cancer with BRCA mutations and/or a homologous recombination deficiency phenotype^13,38^. In addition, some studies have found that the inhibitory effects of olaparib do not always depend on BRCA1/2 status in ovarian and breast cancers. The combination of AKT inhibitors, Topo I inhibitors, and regulators of homologous recombination has clear therapeutic potential in pre-clinical models^39,40^. The anti-tumorigenic activities of ONC201 have been extensively studied in pre-clinical models as well as clinical trials^25^. In understanding its unique mechanism on inhibition of tumor growth, ONC201 has exhibited effective synergy in combination with chemotherapeutic drugs and other small-molecule inhibitors that work by complementary mechanisms^41–43^. ONC206, a derivative and chemical analogue of ONC201, demonstrates enhanced noncompetitive DRD2 antagonism and bioavailability compared to ONC201 and is currently being assessed in a first-in-human trial^26,30,44^. Our previous studies demonstrated that ONC206 caused apoptosis and inhibited tumor growth in a transgenic mouse model of EC through inhibition of the AKT/mTOR pathway. Since inhibition of the AKT/mTOR pathway leads to downregulation of BRCA1/2 and homologous recombination and olaparib activates AKT/mTOR signaling^15,19,45^, the combination of olaparib and ONC206 may have a synergistic effect in inhibiting tumor growth in EC via dual inhibition of the AKT/mTOR pathway. In addition, the unique pharmacological properties and robust safety profile of ONC206 also provide opportunities to combine with other chemotherapeutics and targeted therapies, including olaparib, without additional toxicities for patients. In this study, we have demonstrated a synergistic relationship between olaparib and ONC206 with combination treatment condition *in vitro* and *in vivo*, resulting in inhibition of cell proliferation, induction of apoptosis and cellular stress, induction of DNA damage, and reduction in tumor growth in both obese and lean mice. These results suggest that the olaparib could improve clinical benefit in combination with ONC206 in EC patients.

Somatic mutation or deletion of the PTEN tumor suppressor is the most frequent genetic alteration in endometrioid EC^33^. PTEN is a major negative controller for the down regulation of the PI3K/Akt pathway that controls cell proliferation, growth, survival, and genomic instability^33,46^. Whether PI3K/Akt activity affects the sensitivity of PARP inhibitors to inhibit tumor growth is still controversial, with response in some but not all cancer cell types^13^. It has been reported that loss of PTEN function results in higher response of PARP inhibitors through regulation of homologous recombination repair in EC cells^20,47^. However, recent studies found that mutated PTEN or PTEN-deficiency in EC cell lines did not predict the sensitivity to olaparib in regard to inhibition of cell proliferation^19,37^. Other studies have found that PTEN status may influence the response of EC cells to combined PARP inhibitors and PI3K inhibitors but not PARP inhibitors alone^19^. In this study, we used PTEN mutated ECC-1 cells and PTEN wild-type HEC-1A cells to evaluate the efficacy of olaparib alone and in combination with ONC206^19,48^. The results showed that olaparib and ONC206 inhibited cell proliferation in both cell lines, whereas ECC-1 cells showed higher sensitivity to olaparib, indicating that PTEN status and activity of the PI3K/AKT pathway are relevant to the response to olaparib but not to ONC206. The combination of low doses of olaparib and ONC206 synergized to achieve anti-proliferative effects in both cell lines, regardless of PTEN status. Moreover, combination treatment of olaparib and ONC206 for 4 weeks synergistically inhibited tumor growth in our *Lkb1*^*fl/fl*^ *p53*^*fl/fl*^ transgenic mouse model of endometrioid EC without obvious toxicity, implying that this combination treatment may have inhibitory effects in EC patients with wild type PTEN or PTEN loss tumors.

To better understand the functional mechanisms of olaparib and ONC206 in inhibiting EC cell proliferation and tumor growth, we explored the effects of both drugs and the combination on apoptosis and cellular stress. Induction of apoptosis via TRAIL and activation of the integrated stress response via ClpP are known mechanisms of imipridones that have been previously demonstrated in EC^24,27,29,36,49^. Similarly, PARP inhibitors also lead to induction of apoptosis by inducing ROS and increasing non-reparable DNA damage and expression of FAS and TRAIL-related death receptor 5^50^. Monotherapy with both olaparib and ONC206 resulted in increased cellular stress and cleaved caspase-3, −8 and −9 activities compared to controls. The combination of both drugs led to significant increases in both caspase activities and cellular stress as well as in anti-apoptotic proteins and cellular stress-related proteins including BCL-2, MCL-1, PERK, BIP, and calnexin. Although slightly increased gamma-H2A× protein levels were observed in both cell lines following olaparib and ONC206 treatment, the combination of olaparib and ONC206 significantly increased phosphorylated γ-H2A× expression compared to monotherapy or control in EC cells and in endometrial tumors of the *Lkb1*^*fl/fl*^ *p53*^*fl/fl*^ mouse model. These results imply that decreased DNA repair and increased activity of apoptosis may contribute to the anti-proliferative effects of the olaparib/ONC206 combination treatment in EC cells and endometrial tumors.

Targeting the PI3K/AKT pathway may be another underlying mechanism of combination treatment with olaparib and ONC206 to improve the synergistic inhibition of cell proliferation and tumor growth in EC. This pathway exerts a profound impact on downregulation of BRCA1 and BRCA2, the process of DNA damage repair, and the maintenance of genome stability. Activation of the PI3K/AKT pathway is associated with acquired resistance to PARP inhibitor treatment and may compromise the efficacy of PARP inhibitors^19,51,52^. PTEN-deficient tumors present an enhanced sensitivity to the combination of PI3K and PARP inhibition in EC cells and in a PTEN-deficient mouse model of EC^19,53^. The effect of PARP inhibitors on the PI3K/AKT pathway remains elusive. Olaparib has been shown to reduce phosphorylation of PI3K and AKT in gastric adenocarcinoma cells via the ClC-3/SGK1 regulatory axis^54^. In BRCA-proficient triple negative breast cancer cells, treatment with olaparib did not show significant regulation in the expression and phosphorylation of AKT, but targeting of PI3K activity by BKM120 synergistically increased sensitivity to olaparib in inhibition of cell proliferation^55^. Interestingly, recent studies have also found that olaparib can increase phosphorylation of AKT and activate PI3K/AKT pathway in tumor cells and mouse tumor models, resulting in compromised efficacy of olaparib^19,45,56^. Thus, targeting both PI3K/AKT and PARP may lead to improved anti-tumor activity of PARP inhibitors. Our previous studies have confirmed that ONC201 and ONC206 effectively inhibited the PI3K/AKT/mTOR pathway in EC cells^27,29,36^. In this study, we found that the combination of olaparib and ONC206 showed significant effects on the AKT/mTOR/S6 pathway compared to ONC206 or olaparib alone. The finding of increased inhibition of the PI3K/AKT/mTOR pathway with the combination therapy is consistent with prior studies evaluating this treatment strategy^15,45,51,57^.

Given the role of obesity in the pathogenesis of EC and effects of estrogen and hyperinsulinemia on the PI3K/AKT/mTOR pathway^3^, we explored the effects of olaparib and ONC206 treatment on obese versus lean mice using our *Lkb1*^*fl/fl*^*p53*^*fl/fl*^ model. Tumor weights were significantly increased in the obese mice, consistent with our previous work showing that a high fat diet promotes tumor growth in this model^36,58,59^. ONC201 and ONC206 have been shown to decrease endometrial tumor growth in both obese and lean mice, with suggestion of possible greater effects in obese mice^36^. Correspondingly, olaparib effectively reduced tumor weight in obese and lean mice, and exhibited a more effective inhibitory effect in obese mice in this study. Combination treatment with olaparib and ONC206 was effective in both obese and lean mice with a 91% reduction in tumor weight in obese mice and an 85% reduction in tumor weight in lean mice without any observed side effects. ONC206 has exhibited an excellent safety profile in the clinical trials, and our results suggest that the combination of olaparib and ONC206 should be well tolerated per this pre-clinical mouse study.

In conclusion, treatment with olaparib in combination with ONC206 resulted in synergistic inhibition of EC cell proliferation and reduction in EC tumor weight under obese and lean conditions. Combination treatment increased apoptosis and cellular stress compared to treatment with either drug alone. Dual therapy also led to inhibition of the PI3K/AKT/mTOR pathway. A first-in-human trial of ONC206 is currently underway in central nervous system malignancies. These preclinical studies provide rationale for further exploration of this novel dual therapy of olaparib and ONC206 in clinical trials in EC.

## Methods

### Cell culture and reagents

Two human EC cell lines were used for all experiments: ECC-1 and HEC-1A. ECC-1 cells were maintained in RPMI supplemented with 5% FBS and penicillin-streptomycin. HEC1 cells were maintained in McCoy’s 5A medium with 10% FBS and penicillin-streptomycin. The cells were cultured in a humidified incubator with 5% CO_2_ at 37°C. ONC206 was obtained from Oncoceutics/Chimerix (Philadelphia, PA). Olaparib was obtained from MedChemExpress LLC (Monmouth Junction, NJ). Reagents were purchased from Sigma-Aldrich (St. Louis, MO). Antibodies for western blot analysis were purchased from Cell Signaling technology (Danvers, MA), Santa Cruz Biotechnology (Santa Cruz, CA), and Abcam (Cambridge, MA).

### MTT assay

The effects of ONC206, olaparib, and combination treatment were assessed using the MTT assay. The cell lines were grown in 96-well plates at a concentration of 4000–6000 cells/well for 24 h. The cells were then treated with varying doses of olaparib and ONC206 individually and in combination for a period of 72 h. Five-microliter MTT dissolved in PBS (5 mg/ml) was added to each well. After an additional hour of incubation, the supernatant was discarded, and the reaction was terminated with the addition of 100 µl DMSO. MTT was quantified by measuring the light absorbance at 570 nm using the Tecan microplate reader (Morrisville, NC). The Quest Graph IC50 Calculator (AAT Bioquest, Sunnyvale, CA) was used to calculate the IC50s for each drug. The combination index (CI) was calculated by the Bliss Independence model to determine whether the drug effects were additive (CI = 1), synergistic (CI < 1), or antagonistic (CI > 1)^60^. Each experiment was performed in triplicate and repeated for consistency of results.

### Cleaved caspase-3, -8, and -9 assays

Apoptosis was assessed following treatment with ONC206, olaparib, the combination, or vehicle using the colorimetric cleaved caspase-3, -8, and −9 assays (AAT Bioquest, Sunnyvale, CA). The ECC-1 and HEC1A cells were plated in 6-well plates at a concentration of 2.5 × 10^5^. After incubation for 24 h, both cell lines were treated with different concentrations of olaparib, ONC206, the combination, or vehicle. After 12 h of treatment, 180 µl of caspase lysis buffer was added to each well. Protein concentration was determined using the BCA assay. Lysates were then seeded on black 96-well plates and incubated with caspase buffer and DEVD substrate. A Tecan microplate reader was then used to determine the fluorescence of each well: Ex/Em = 400/505 nm for cleaved caspase-3, Ex/Em = 376/482 for cleaved caspase-8, and Ex/Em = 341/441 for cleaved caspase-9. Each experiment was performed in triplicate and repeated for consistency of results.

### Reactive oxygen species (ROS) assay

ROS was evaluated using the DCFH-DA assay to measure reactive oxygen species. Cells were plated on 96-well plates and incubated for 24 h. The cells were then treated with olaparib, ONC206, the combination, or vehicle for 16 h. The oxidation-sensitive probe DCFH-DA was then added (10 µM) to each well for 30 min. Fluorescence was measured at an excitation wavelength of 485 nm and an emission wavelength of 530 nm using a Tecan plate reader. Each experiment was performed in triplicate and repeated for consistency of results.

### JC-1 assay

Mitochondrial membrane potential was analyzed with the specific fluorescent probe JC-1 (AAT Bioquest, Sunnyvale, CA). The ECC-1 and HEC-1A cells were plated in 96 well plates overnight and treated with olaparib, ONC206, the combination, or vehicle for 16 h. The cells were then treated with 2 µM JC-1 for 30 min at 37°C. The microplate reader measured the fluorescence intensity at the Ex/Em = 480/590 nm for the JC-1 assay. Each experiment was repeated three times to assess for consistency of results.

### Western immunoblotting

The ECC-1 and HEC-1A cells were seeded in 6-well plates at a concentration of 2.5 × 10^5^ and incubated for 24 h. The cells were then treated with olaparib, ONC206, the combination, or vehicle for 18–36 h. After treatment, 180 µl of RIPA buffer was added to each well, and cell lysates were collected. Protein concentration was determined using the BCA assay. Equal amounts of protein were denatured at 95°C, separated by gel electrophoresis, and transferred to a PVDF membrane. Membranes were blocked with 5% nonfat dry milk and then incubated with primary antibodies overnight at 4°C. The membranes were then washed and incubated with the appropriate peroxidase-conjugated secondary antibody for 1 h. Antibody-bound proteins were then detected using the ChemiDoc Image System (Bio-Rad, Hercules, CA). The membranes were then stripped and re-probed using B-actin or a-tubulin antibodies to confirm equal protein loading. Primary antibodies used in this study included BCL-2, MCL-1, PERK, IRE, calnexin, phosphorylated (phos)-S6, pan-S6, phos-AKT, pan-AKT, a-tubulin, and B-actin. Experiments were repeated three times to confirm consistent results.

### *Lkb1*^*fl/fl*^*p53*^*fl/fl*^ mouse model of endometrioid EC

The *Lkb1*^*fl/fl*^*p53*^*fl/fl*^ genetically engineered mouse model of endometrioid EC was used in this study^58^. Given that obesity is a driver of EC and results in more aggressive tumor behavior in our mouse model, we decided to look at the effects of olaparib and ONC206 in both lean and obese mice as we have in prior studies^36,59^. To model diet-induced obesity, mice were treated with either a low fat diet (consisting of 10% of calories from fat, “lean”) or high fat diet (consisting of 60% of calories from fat, “obese”) at 3 weeks of age. At 6–8 weeks of age, mice were injected with adenovirus Ad5-CMV-Cre (AdCre, Transfer Vector Core, University of Iowa) into one uterine horn. Mice were divided into eight groups—four lean and four obese with ten mice in each group. The mice were treated by oral gavage with either placebo, ONC206 at a dose of 100 mg/kg weekly, olaparib 25 mg/kg daily, or the combination. All mice were euthanized after 4 weeks of treatment. Tumor tissues and blood samples were collected and stored at −70°C. The protocol for the animal experiments was reviewed and approved by the Institutional Animal Care and Use Committee at the University of North Carolina at Chapel Hill.

### Immunohistochemistry

Tumors from the *Lkb1*^*fl/fl*^*p53*^*fl/fl*^ mice were formalin-fixed and paraffin embedded using standard methodologies at the Animal Histopathology Core Facility at the University of North Carolina at Chapel Hill. Slides (5 µm) were stained with anti-Ki-67 and anti-H2A×. Slides were then scanned using Motic (Houston, TX) and scored by ImagePro software (Vista, CA).

### Statistical analysis

Descriptive statistics were used to summarize data. Student’s t-test and ANOVA were used for parametric data, and Wilcoxon and Kruskal-Wallis were used for non-parametric data to evaluate statistical significance between treatment groups. Differences were considered significant if the p-value was less than 0.05. GraphPad Prism 6 (La Jolla, CA) was used for all graphs and significance tests.

## Data Availability

All data generated or analyzed during this study are included in this article. The datasets used and/or analyzed during the current study are available from the corresponding authors on reasonable requests.
